# Use of Involuntary Emergency Treatment by Physicians and Law Enforcement for Persons With High-Risk Drug Use or Alcohol Dependence

**DOI:** 10.1001/jamanetworkopen.2021.20682

**Published:** 2021-08-13

**Authors:** Kathleen E. Coffey, Audrey Aitelli, Michael Milligan, Andrzej Niemierko, Thomas Broom, Helen A. Shih

**Affiliations:** 1 Boston Municipal Court, West Roxbury Division, Jamaica Plain, Massachusetts; 2 Department of Radiation Oncology, Massachusetts General Hospital, Boston

## Abstract

This cohort study evaluates the number of petitions for temporary involuntary commitment of individuals who pose a risk to themselves or others after implementation of a pilot program to streamline the process in Boston, Massachusetts.

## Introduction

In 1986, the state of Massachusetts enacted section 35 of chapter 123 of the Massachusetts General Law, commonly known as Section 35, to allow for the temporary involuntary commitment of individuals who pose a risk to themselves or others secondary to substance use.^1^ Historically, the Section 35 process required multiple in-person court appearances by petitioners; therefore, this process was not frequently used by busy physicians and police officers. In May 2017, the West Roxbury Division of the Boston Municipal Court initiated a pilot program to streamline the Section 35 process, in which faxed affidavits were accepted from physicians and police officers in lieu of in-person court appearances. The purpose of this study was to assess how the pilot program has altered the use of the Section 35 process and to examine the social and clinical characteristics of individuals who were involuntarily committed to substance use rehabilitation programs.

## Methods

In this cohort study, we compared the number of public court records of all Section 35 petitions filed in Boston, Massachusetts, between 2016 and 2018. The outcome of each petition was recorded, and affidavits and court-prepared medical reports were used to collect demographic and clinical data on individuals who were involuntarily committed through the West Roxbury Court. All data were collected between May 2019 and April 2021, and analyses were performed between November 2020 and April 2021. Legal criteria for commitment are provided in the eAppendix in the Supplement. We compared the number of Section 35 petitions filed before and after implementation of the pilot program to evaluate whether there was an association between program implementation and an increase in the number of petitions filed. This study followed the Strengthening the Reporting of Observational Studies in Epidemiology (STROBE) reporting guideline. This study was deemed exempt from review and the requirement for informed consent was waived by the Mass General Brigham Institutional Review Board because publicly available data were used. Descriptive statistics were used to analyze the data; data analysis was completed with Excel version 2102 (Microsoft).

## Results

A total of 213 individuals (142 men [66%] and 71 women [30%]) with a mean (SD) age of 43.8 (13.0) years underwent temporary involuntary commitment secondary to substance use under Section 35 of the Massachusetts General Law. The number of Section 35 petitions filed in the West Roxbury Court increased 272.3%, with 112 petitions filed in 2016 and 417 filed in 2018. During the study period (2016-2018), the number of Section 35 petitions filed across all other jurisdictions in Boston, Massachusetts, increased by 30.1%, with 658 petitions filed before program implementation and 856 filed after implementation (Figure). The increase observed in the West Roxbury Court can be attributed to the number of petitions filed by physicians and police officers, which increased from 11 of 112 (9.8%) petitions filed in 2016 to 274 of 417 petitions in 2018 (65.7%). The number of unique physicians and police officers filing Section 35 petitions increased over time, with 7 physicians and 1 police officer filing petitions in 2016, 42 physicians and 18 police officers filing petitions in 2017, and 77 physicians and 26 police officers filing petitions in 2018. In 2018, 170 of 274 petitions (62.0%) in the West Roxbury Court involved 138 unique individuals and led to involuntary commitment to substance use rehabilitation programs.

**Figure.**
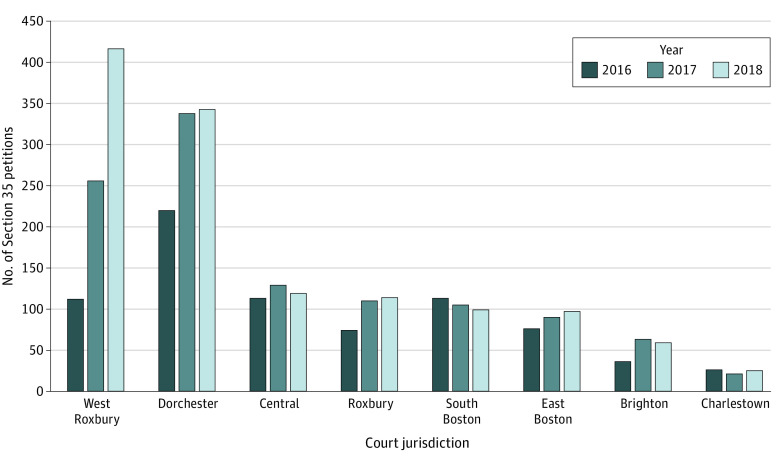
Section 35 Petitions Filed in the Municipal Court Jurisdictions of Boston, Massachusetts, 2016 to 2018 In May 2017, the West Roxbury Court initiated its pilot program to streamline the Section 35 process by allowing physicians and police officers to provide written affidavits to support their petitions, rather than requiring in-person court appearances. The Dorchester Court began a more limited program in 2017 in which it accepted written affidavits from physicians but continued to require in-person court appearances for petitioning police officers.

The Table presents the demographic and clinical characteristics of these individuals. The most frequently used substances included alcohol (104 of 138 individuals [75.4%]) and opioids (72 of 138 individuals [52.2%]). The proportions of individuals with comorbid mental health diagnoses, those who previously sought treatment for substance use disorder, and those with hospitalizations in the month before commitment were greater than 60%. Notably, the proportion of individuals committed to substance use rehabilitation programs through the Section 35 program who were reported to be homeless increased from 3 of 8 (37.5%) in 2016 to 84 of 138 (60.9%) in 2018.

**Table.** Section 35 Petitions and Commitments Filed in West Roxbury Court and Demographic and Clinical Characteristics of Individuals Involuntarily Committed to Substance Use Rehabilitation Programs^a^

Petition, commitment, or characteristic	Year		
	2016	2017	2018
**Petitions and commitments, No.**			
Petitions filed by physicians or police	11	123	274
Unique individuals petitioned	9	98	187
Total commitments	9	73	170
Unique individuals committed	8	67	138
**Demographic and clinical characteristics of unique individuals committed**			
Substance use			
Alcohol	6 (75.0)	49 (73.1)	104 (75.4)
Opioids	4 (50.0)	32 (47.8)	72 (52.2)
Stimulants	5 (62.5)	28 (41.8)	61 (44.2)
Other sedatives	3 (37.5)	13 (19.4)	37 (26.8)
Cannabinoids	2 (25.0)	16 (23.9)	33 (23.9)
Hallucinogens	0	2 (3.0)	6 (4.3)
Age group, y			
30 or younger	1 (12.5)	13 (19.4)	36 (26.1)
31-50	5 (62.5)	22 (32.8)	52 (37.7)
51 or older	2 (25.0)	32 (47.8)	50 (36.2)
Sex			
Male	5 (62.5)	47 (70.1)	90 (65.2)
Female	3 (37.5)	20 (29.9)	48 (34.8)
Race/ethnicity			
Non-Hispanic White	6 (75.0)	47 (70.1)	99 (71.7)
Non-Hispanic Black	1 (12.5)	15 (22.4)	19 (13.8)
Hispanic	1 (12.5)	4 (6.0)	17 (12.3)
Other^b^	0	1 (1.5)	3 (2.2)
Mental health diagnoses per medical report			
Any mental health diagnosis	5 (62.5)	45 (67.2)	92 (66.7)
Depression	3 (37.5)	24 (35.8)	57 (41.3)
Anxiety	1 (12.5)	17 (25.4)	41 (29.7)
Bipolar disorder	1 (12.5)	15 (22.4)	18 (13.0)
Posttraumatic stress disorder	1 (12.5)	15 (22.4)	26 (18.8)
Suicidal ideation/intention	3 (37.5)	18 (26.9)	49 (35.5)
No. of hospital visits in the previous month			
None	3 (37.5)	26 (38.8)	64 (46.4)
1-2	3 (37.5)	19 (28.4)	23 (16.7)
3-4	0	9 (13.4)	18 (13.0)
≥5	2 (25.0)	13 (19.4)	33 (23.9)
Housing			
Homeless	3 (37.5)	47 (70.1)	84 (60.9)
Stable housing	3 (37.5)	18 (26.9)	34 (24.6)
Unknown	2 (25.0)	2 (3.0)	20 (14.5)
Sought treatment for substance use or substance use disorder			
Yes	5 (62.5)	52 (77.6)	94 (68.1)
No	3 (37.5)	15 (22.4)	44 (31.9)
Previous Section 35 commitment			
Yes	3 (37.5)	30 (44.8)	47 (34.1)
No	5 (62.5)	37 (55.2)	91 (65.9)
Previous overdose			
Yes	4 (50.0)	31 (46.3)	58 (42.0)
No	4 (50.0)	36 (53.7)	80 (58.0)

^a^ Unless otherwise specified, data represent the No. (%) of individuals.

^b^ In the data from 2017, the other category includes individuals with unknown race/ethnicity. In the 2018 data, the other category includes 1 Asian individual and 2 individuals with unknown race/ethnicity.

## Discussion

The social and medical complexities inherent in treating individuals with substance use disorders require collaboration among medical practitioners, law enforcement, and the courts. Removing the requirement of in-person court appearances for physicians and police was followed by a 272.3% increase in petitions for substance use–related involuntary commitment in a single jurisdiction. To be ethically permissible, involuntary commitments in the setting of impaired capacity must maximize benefits to the individual while minimizing potential harms.^2^ The high proportion of individuals with comorbid mental health diagnoses seen in this cohort suggests that optimal treatment should involve immersive, dual-diagnosis treatment. The number of individuals experiencing homelessness increased over the course of the study period, which suggests that the pilot program extended treatment to individuals with fewer social supports. Although this study is limited by lack of longitudinal follow-up and focuses on a single jurisdiction in Boston, Massachusetts, the findings suggest the need to further explore a multidisciplinary approach to caring for individuals with substance use disorders.

## Notes

Supplement. **eAppendix.** Legal Criteria for Involuntary Commitment Under the Section 35 Process in Massachusetts
