# Supplementary material for: Use of Involuntary Emergency Treatment by Physicians and Law Enforcement for Persons With High-Risk Drug Use or Alcohol Dependence
Source: JAMA Netw Open. 2021 Aug 13;4(8):e2120682. doi: 10.1001/jamanetworkopen.2021.20682 (PMC8363915; doi:10.1001/jamanetworkopen.2021.20682)
Supplement: Supplement. — eAppendix. Legal Criteria for Involuntary Commitment Under the Section 35 Process in Massachusetts [file jamanetwopen-e2120682-s001.pdf]

Supplemental Online Content

Coffey KE, Aitelli A, Milligan M, Niemierko A, Broom T, Shih HA. Use of involuntary emergency treatment by physicians and law enforcement for persons with high-risk drug use or alcohol dependence. *JAMA Netw Open*. 2021;4(8):e2120682.
doi:10.1001/jamanetworkopen.2021.20682

eAppendix. Legal Criteria for Involuntary Commitment Under the Section 35 Process in Massachusetts

This supplementary material has been provided by the authors to give readers additional information about their work.

© 2021 Coffey KE et al. *JAMA Network Open*.

eAppendix. Legal Criteria for Involuntary Commitment Under the Section 35 Process in Massachusetts

To issue an order of commitment under M.G.L. ch. 123 sec. 35, a judge must find by clear and convincing evidence, that (1) the person whose commitment is sought is an individual with an alcohol or substance use disorder and (2) there is a likelihood of serious harm as a result of the person's alcohol or substance use disorder. There are three different paths by which a judge may find a 'likelihood of serious harm.'

(1) A substantial risk of physical harm to the person as manifested by evidence of, threats of, or attempts at, suicide or serious bodily harm,
(2) A substantial risk of physical harm to other persons as manifested by evidence of homicidal or other violent behavior or evidence that others are placed in reasonable fear of violent behavior and serious physical harm,
(3) A very substantial risk of physical impairment or injury to the person himself as manifested by evidence that such person's judgement is so affected that he is unable to protect himself in the community and that reasonable provision for his protection is not available in the community.

The harm must be 'imminent' which means a substantial risk that the harm will materialize in the reasonably short term in days or weeks rather than in months. See: *In the Matter of a Minor,484 Mass. 295 (2020)*

© 2021 Coffey KE et al. *JAMA Network Open*.
